# General Overview of *Klebsiella pneumonia*: Epidemiology and the Role of Siderophores in Its Pathogenicity

**DOI:** 10.3390/biology13020078

**Published:** 2024-01-27

**Authors:** Rim Abbas, Mohamed Chakkour, Hiba Zein El Dine, Eseiwi Folorunsho Obaseki, Soumaya T. Obeid, Aya Jezzini, Ghassan Ghssein, Zeinab Ezzeddine

**Affiliations:** 1 Faculty of Health Sciences, Beirut Arab University, Beirut P.O. Box 11-5020, Lebanon; 2 Department of Biological Sciences, Wayne State University, Detroit, MI 48202, USA; mohamedchakkour@gmail.com (M.C.);; 3 Department of Cell Physiology and Metabolism, Faculty of Medicine, University of Geneva, 1205 Geneva, Switzerland; hiba.zeineldine@unige.ch; 4 Laboratory Sciences Department, Faculty of Public Health, Islamic University of Lebanon (IUL), Khalde P.O. Box 30014, Lebanon

**Keywords:** *Klebsiella pneumoniae*, pathogenesis, epidemiology, siderophores

## Abstract

**Simple Summary:**

Numerous diseases, such as liver abscesses, bacteremias, pneumonia, and urinary tract infections, are caused by *Klebsiella pneumonia* (*K. pneumonia*). Historically, immunocompromised patients have been the main target of serious infections induced by *K. pneumoniae*. However, with the recent appearance and dissemination of hypervirulent strains, healthy individuals have also become susceptible to infection. Moreover, treating *K. pneumoniae* infections is extremely difficult due to the strains’ increased antibiotic resistance. This review summarizes the various virulence factors of this bacterium, especially metallophores. The bacteriology, pathology, and epidemiology of *K. pneumonia* are also included in this review.

**Abstract:**

The opportunistic pathogen *Klebsiella pneumoniae* (*K. pneumoniae*) can colonize mucosal surfaces and spread from mucosae to other tissues, causing fatal infections. Medical equipment and the healthcare setting can become colonized by *Klebsiella* species, which are widely distributed in nature and can be found in water, soil, and animals. Moreover, a substantial number of community-acquired illnesses are also caused by this organism worldwide. These infections are characterized by a high rate of morbidity and mortality as well as the capacity to spread metastatically. Hypervirulent *Klebsiella* strains are thought to be connected to these infections. Four components are critical to this bacterium’s pathogenicity—the capsule, lipopolysaccharide, fimbriae, and siderophores. Siderophores are secondary metabolites that allow iron to sequester from the surrounding medium and transport it to the intracellular compartment of the bacteria. A number of variables may lead to *K. pneumoniae* colonization in a specific area. Risk factors for infection include local healthcare practices, antibiotic use and misuse, infection control procedures, nutrition, gender, and age.

## 1. Introduction

*Klebsiella pneumoniae* (*K. pneumoniae*), a Gram-negative bacterium, is recognized as an opportunistic pathogen capable of causing a wide range of infections in humans. Traditionally, *K. pneumoniae* has been linked to bacteremia, pneumonia, and urinary tract infections (UTIs) in immunocompromised or often hospitalized individuals [1]. In addition, *K. pneumoniae* isolates that belong to the carbapenem-resistant Enterobacteriaceae (CRE) family are characterized by their resistance to carbapenems, a class of commonly used wide-spectrum antibiotics. These isolates have been identified by the World Health Organization (WHO) as a “critical concern”, meaning that there is a serious need for the research and development of new antibiotics to target this significant threat to human health [2]. In fact, *K. pneumoniae* is the major cause of nosocomial pneumonia and one of the few Gram-negative rods that can cause primary pneumonia. Classical *K. pneumonia* (cKP) is characterized by nosocomial infections, which are most common in elderly or immunodeficient patients [3]. Over the recent decades, a unique strain of *K. pneumoniae*, known as hypervirulent *K. pneumoniae* (*hvKp*), has emerged, causing infections among both healthy and immunocompromised individuals. In contrast to cKP, the hypervirulent strain frequently infects healthy people [4] and is mostly linked to high pathogenicity and mortality rates [5]. Liver abscess in healthy individuals is the primary clinical manifestation of *hvKp* infection, in addition to several infections, including pneumonia, meningitis, and endophthalmitis [6]. The ability of *hvKp* to spread metastatically from the site of infection in an immunocompetent host sets it apart from cKP and other Enterobacteriaceae members [7]. Most *hvKp* strains have proven to be quite responsive to antibiotics thus far. However, it seems that the *hvKp* phenotypes and the antibiotic-resistant cKP phenotypes have already started to merge. According to previous findings, certain *hvKp* strains have developed the ability to produce carbapenemases and extended-spectrum β-lactamases (ESBLs), allowing *hvKp* to become an antibiotic-resistant strain [8,9].

The virulence factors in *hvKp* are lipopolysaccharides (LPS), siderophores, capsules, and fimbriae [10]. Among these, siderophores and small iron-binding metabolites [11] are of particular interest. Iron is an essential metal ion for bacterial growth, and its bioavailability is typically below the required micromolar concentration [12]. Free iron (Fe^3+^) is scarce under physiological conditions since it is bound to transferrin and heme during infection. Iron absorption in vivo by bacteria often requires a siderophore-dependent iron acquisition system [13]. These molecules, produced and secreted by bacteria, chelate available iron in the surrounding environment and import it into the cells. For this reason, siderophores are frequently regarded as essential virulence factors for bacterial survival and pathogenesis [14].

Once iron-loaded, certain outer membrane receptors identify iron siderophore complexes and transfer them to the periplasm, where they integrate with periplasmic binding proteins that transport them to the inner membrane. Eventually, iron enters the cytoplasm through an ATP-binding cassette (ABC) transporter, where it is converted from ferric iron to ferrous iron, the form used by the pathogen [15]. Previous research has demonstrated that when compared to cKP, the hypervirulent strain exhibits a distinctive 6- to 10-fold increase in siderophore production [14], namely enterobactin (Ent), salmochelin (Iro), yersiniabactin (Ybt), and aerobactin (Iuc). These four siderophores increase the bacterial growth efficiency [1]. However, human neutrophils and epithelial cells restrict iron assimilation via Ent by secreting lipocalin-2 (Lcn2), which binds to and prevents bacteria from taking up iron-loaded Ent [16]. Ent is the principal siderophore also secreted by several less pathogenic Enterobacteria. The modified Ent, which is not bound by siderocalin, is called salmochelin. It is not only used by *Klebsiella* but also by pathogenic strains, including *E. coli* and *Salmonella*. Nevertheless, the other three siderophores are not bound by Lcn2. To account for this inhibition, pathogenic strains have developed an alternative form of Ent (Salmochelin) for iron acquisition during infection. Salmochelin was found to evade sequestration by Lcn2, allowing the pathogenic Gram-negative bacterial strains to evade the host’s innate immunity. Thus, in a mouse sepsis model, this supported bacterial growth in the host organism has been linked to increased virulence [17].

Aerobactin (Iuc), on the other hand, has been long known to be associated with virulence; numerous studies have shown that it plays a critical role in enhanced iron acquisition, bacterial growth, and/or virulence in a variety of murine models, human ascites fluid, and blood [18]. Iuc appears to be the most important factor affecting virulence in vitro and in vivo, even in strains that carry all four siderophore-encoding loci [19]. It is also considered a useful biomarker for detecting hypervirulent isolates [20]. This review provides insights into *K. pneumoniae* pathophysiology and summarizes all aspects related to the four bacterial siderophores: biosynthesis, export, import, and genetic regulation.

## 2. Bacteriology of *K. pneumoniae*

*K. pneumoniae* belongs to the Gram-negative Enterobacteriaceae family, along with several common pathogens, such as *Escherichia coli*, *Salmonella*, and *Shigella* [21]. It is a rod-shaped, non-motile, and non-spore-forming bacterium, ranging from 0.3 to 2.0 μm in width and 0.6 to 6.0 μm in length, with a distinctive mucoid appearance on agar plates. As a facultative anaerobe, *K. pneumoniae* is adapted to thrive in both aerobic (presence of oxygen) and anaerobic (absence of oxygen) environments. Biochemically, it is lactose-fermenting, catalase-positive, and cytochrome oxidase-negative.

A hallmark feature of *K. pneumoniae* is its encapsulated morphology. The bacterium produces a pronounced extracellular polysaccharide layer, a capsule, which encloses the cell structure and acts as a shield against host innate immunity [22]. Capsules are known to promote immune evasion by hindering host clearance through phagocytosis [23], enhancing bacterial resistance to intracellular killing [24], and antigenically mimicking host glycans. The presence of a capsule increases bacterial survival, and its loss makes *K. pneumoniae* less or nonvirulent [25]. Capsule production is controlled by a single capsule polysaccharide (cps) locus, which harbors several genes [26]. To date, *K. pneumoniae* is known to produce at least 79 types of capsules [26], which differ from one another by the structure and components of the repeating polysaccharide unit in the capsular polysaccharide [22]. Unlike cKP, *hvKp* strains can produce hypercapsules through several specific virulence genes, such as *c-rmpA*, *c-rmpA2*, *p-rmpA*, *p-rmpA2*, and *wzy-K1* [19]. This phenotype is notably found in K1 and K2 serotypes and is linked to hypervirulence [27].

As a Gram-negative bacterium, both cKp and *hvKp* have lipopolysaccharides (LPS) in their outer membrane, also termed endotoxin. LPS consists of lipid A, a core oligosaccharide, and O-antigens that serve as a protective layer against complement-mediated killing [28]. To date, there are at least 8 O-antigen serotypes in *K. pneumoniae*, with the O1 antigen being the most common among clinical strains.

Moreover, two types of fimbriae are widespread in *K. pneumoniae* strains [29]: Type 1 and 3 fimbriae, which are encoded by the *fim* and *mrkABCD* operons, respectively, and aid in bacterial adhesion and invasion of host cells and biofilm formation.

## 3. Pathophysiology of *K. pneumoniae*

The common opportunistic *K. pneumoniae* greatly affects individuals with compromised immunity or whose immunity is weakened by other infections. However, the very invasive hypervirulent *K. pneumoniae* is capable of infecting healthy individuals, leading to community-acquired infections, including pyogenic liver abscess, meningitis, necrotizing fasciitis, endophthalmitis, and severe pneumonia [6]. The development of hospital-acquired infection is usually preceded by gastrointestinal (GI) tract colonization by *K. pneumoniae*. Additionally, this colonization can further extend to the urinary tract, respiratory tract, and bloodstream [30]. Another infectious aspect of *K. pneumoniae* is its biofilm, which can develop on medical equipment (catheters and endotracheal tubes), causing a substantial infection source in catheterized patients [31]. Furthermore, *K. pneumoniae* nosocomial infections tend to be chronic due to two main factors: The development of immune-evading biofilms in vivo and the manufacturing of enzymes that may inactivate a certain antibiotic, making the organism resistant to this antibiotic, such as the extended-spectrum β-lactamases or carbapenemases. The production of antibiotic-resistance enzymes makes it nearly impossible for physicians to find an effective antibiotic to treat infected patients [32].

After entering the human body, *K. pneumoniae* can colonize the respiratory tract, infect the lungs, and cause severe pneumonia. To establish a prominent infection, one of the major strategies of *K. pneumoniae* is to evade the host’s immunity, particularly via conferring resistance to phagocytosis by immune cells [23]. Several virulent factors contributing to the pathophysiology of *K. pneumoniae* have been studied extensively. These factors include the production of capsular polysaccharides (CPSs), lipopolysaccharides (LPSs), fimbriae, outer membrane proteins (OMPs), and iron-binding siderophores [33]. The CPS is considered the primary virulence factor of *K. pneumonia*; it is an acid polysaccharide composed of three to six repeating units of sugar. Its synthesis involves the Wzy-dependent polymerization pathway and is controlled by the *cps* gene cluster [34,35]. Glycosyltransferases catalyze the assembly of single sugar-repeat units, initiating CPS synthesis, which is then transported to the inner cell membrane where it undergoes Wzy-dependent polymerization and export to the cell surface [36]. After crossing the epithelial barrier, pathogens become prone to phagocytosis by macrophages, neutrophils, or dendritic cells (DCs). However, the presence of a thick capsule on the surface of *K. pneumoniae* blocks its binding to and internalization by immune cells. In fact, the K1-CPS in the hypervirulent *K. pneumoniae* significantly limits the interaction between the bacteria and macrophages compared with non-hypervirulent strains [37]. In addition, CPS plays an anti-inflammatory role by inhibiting the secretion of IL-8 by respiratory pathway epithelial cells, thus, cutting off any downstream immune response to IL-8. Respiratory tract epithelia express Toll-like receptors (TLRs), which recognize cell surface molecules carried by pathogens and trigger downstream signaling pathways, leading to IL-8 secretion. CPS has the capability to bind TLR2 and TLR4 on pulmonary epithelial cells and block their downstream signaling [36]. Another response to infection by airway epithelial cells is the secretion of several antimicrobial polypeptides to weaken and kill the pathogen. CPS attached to the surface of *K. pneumoniae* forms a shield that protects the bacteria from these antimicrobial molecules, while free CPSs released by the bacteria capture these molecules before reaching the bacterial surface. CPS also inhibits antimicrobial substance secretion by epithelial cells by blocking the TLR-mediated responses [36]. Moreover, *K. pneumoniae* CPS can prevent DC maturation, reducing the number of natural killer cells and blocking their migration to the site of infection [36]. Another role for CPSs in evading immune response is their ability to block the assembly of the complement system components and prevent the subsequent membrane attack complex pores from breaching the bacterial outer membrane [38].

Another virulent factor is the lipopolysaccharide (LPS). LPSs in hypervirulent *K. pneumoniae* are directly linked to increased virulence. Studies showed that LPSs, and particularly the O-antigen, block the complement system components from accessing the bacteria, thus, protecting it from immune complement-mediated destruction [39]. After the onset of bacteremia, O-antigen also stimulates bacterial spread to and colonization of internal organs [39]. Comparatively, lipid A and core polysaccharides provide the bacteria with resistance to antibacterial molecules secreted by the host cells [40] and phagocytosis by macrophages [41]. Furthermore, LPS, and particularly its lipid A component, can induce a strong immune response by activating Toll-like receptor 4 (TLR4), leading to the expression of different cytokines and chemokines and the recruitment of neutrophils and macrophages. However, it is still not clearly established whether the LPS produced by *hvKp* plays a distinctive role in their enhanced virulence.

In addition to CPS and LPS, *K. pneumoniae* has thin surface appendages called fimbriae on their outer membranes. At least four types of experimentally verified fimbriae exist: fimbriae type 1, fimbriae type 3, Kpc fimbriae, and KPF-28 fimbriae [36]. Fimbriae are thin, stiff, adhesive, thread-like projections located on the surface of the bacterial outer membrane. These structures stretch beyond the capsule and facilitate bacterial bonds to mannose-rich structures on host cells or extracellular matrices. Thus, fimbriae play a key role in facilitating bacterial adhesion to and infection spread within internal tissues. Type 1 fimbriae are required by *K. pneumoniae* to establish infection in the urinary tract [42], while type 3 fimbriae mediate the adhesion of *K. pneumoniae* to epithelial cells in kidney and lung tissues [43]. On the other hand, type 3 fimbriae and kpc fimbriae majorly contribute to *K. pneumoniae* biofilm formation [44,45]. It is worth mentioning that biofilm formation is considered a critical virulence trait for many microorganisms, including *K. pneumoniae*. In fact, bacterial biofilms account for 65–80% of bacterial infections, where biofilms provide elevated resistance to host immunity, antimicrobial factors, and exogenic stressors [46]. These short pili structures have been shown to bind to extracellular matrix proteins (such as type IV and V collagens) as well as to abiotic surfaces (such as urinary catheters), revealing their remarkable contribution to biofilm formation [28,31,47]. Experimental evidence shows that KPF-28 fimbriae mediate *K. pneumoniae* attachment to human colon carcinoma cell lines, suggesting the involvement of KFP-28 fimbriae in bacterial colonization in the intestinal tissue [48].

Finally, all the above factors are essential contributors to *K. pneumoniae* virulence, yet there are still more. The bacterial outer membrane proteins (OMPs) are the missing part that completes the virulence puzzle created by all other virulent factors. For instance, OmpA, one of the major outer membrane proteins in *K. pneumoniae*, can attenuate the inflammatory response triggered by airway epithelial cells. This inhibitory role of OmpA is independent of CPS and has an additive effect; thus, CPS alone is not enough to trigger full inhibition of epithelial-inflammatory reaction [49]. OmpA can also prevent phagocytosis by macrophages located in alveolar sacs [41] and resist the cytotoxicity of the host’s antimicrobial molecules [50]. The outer membrane porins are additional essential OMPs. In addition to their role in evading phagocytosis, these porins facilitate the diffusion of nutrients, carbohydrates, and hydrophilic molecules essential for bacterial growth into the cell [51]. Efflux pumps, such as the AcrAB pump in *K. pneumoniae*, form another group of outer membrane proteins. They play critical roles in pumping antibiotics, antimicrobial peptides, and other harmful substances to the outside of the bacteria [52].

In summary, the pathophysiology of bacteria involves mechanisms of survival, development, and virulent infection within a host organism. In other words, the pathophysiology of *K. pneumoniae* is directly linked to its ability to evade the host’s immune system via its CPS and LPS, efficiently attach to and colonize its surroundings with the help of fimbriae and acquire nutrients from its surroundings through OMPs. Nevertheless, iron-binding siderophores (discussed in detail below) contribute hugely to *K. pneumoniae* pathophysiology through their role in iron uptake, which is an indispensable element for bacterial growth and replication.

## 4. Epidemiology of *K. pneumoniae* (Mainly Hypervirulent *K. pneumoniae* in Eastern Asia)

Several factors can result in the colonization of *K. pneumoniae* in a particular region. Risk factors such as regional healthcare practices, use and misuse of antibiotics, infection control measures, nutrition, gender, and age could predispose a community to *K. pneumoniae* infection. As such, the colonization rate varies from country to country. *K. pneumoniae* may exist as part of the normal microbiota in both animal and human hosts. In humans, *K. pneumoniae* coexists with the microbial flora in the intestinal tract and nasopharynx. *K. pneumoniae* in stool samples ranges from 5% to 38%, while the detection rate in nasopharyngeal samples ranges from 1% to 6% [30]. However, hospitalized patients tend to have significantly higher carrier levels, with up to 77% in the stool and 19% in the pharynx [30]. Apart from the contributory factor of the hospital environment, antibiotic misuse practices have also led to a high rate of this colonization [53]. Healthy individuals may harbor cKP colonization, yet infection is uncommon without some degree of host compromise. On the other hand, healthy individuals carrying *hvKp* are significantly more susceptible to infection. The remainder of this section will focus on *hvKp*.

**Figure 1:**
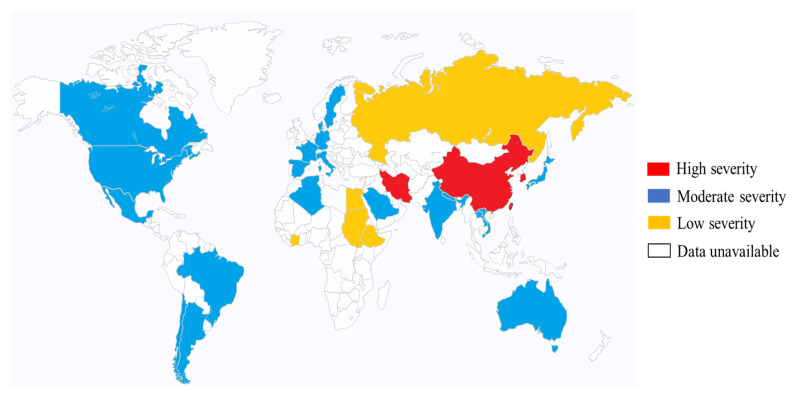
Global distribution of *hvKp*. The severity of *hvKp* is differentiated by color. Regions with high severity have endemic spread of *hvKp*. This manifests as severe clinical outcomes in regions. Moderate severity regions have moderate case studies of *hvKp* infections. Likewise, the low-severity region has few hospital-reported cases of *hvKp* infection [65,66,67,68,69].

Case studies in the 1980s originating from Taiwan were the first to document instances of community-acquired liver abscesses induced by *hvKp* in individuals without underlying health issues, alongside severe concurrent complications, such as meningitis and endophthalmitis [54,55]. Since then, other cases of *hvKp* have increasingly been reported across the globe, possibly due to increasing population migration [56,57,58,59,60,61]. However, the frequency of *hvKp* infection in Asia remains high. For example, in a Chinese study conducted from January 2013 to October 2015, a total of 369 *K. pneumoniae* isolates were consecutively isolated from the specimens of patients with various invasive infections at the First Affiliated Hospital of Wenzhou Medical University located in Wenzhou, east China. The *K. pneumoniae* isolates from the specimens of the respiratory tract, urinary tract, and intestinal tract were excluded in this investigation because it is difficult to discriminate invasive isolates from colonizing isolates. This study identified 22.89% of isolated *K. pneumoniae* strains to be of *hvKp* [62]. This is significantly higher than a report from Spain (conducted in a teaching hospital) between 2007 and 2013 (5.4%, 53/878) and a study in Alberta, Canada (8.2%) [63,64]. A predilection of the *hvKp* infection in Asians has been observed even among Asians residing in Western countries, although the reason for this remains unclear. However, it is evident that there is a growing occurrence of infections caused by *hvKp* in other ethnicities and climes. As such, there is a need for heightened awareness of this condition, which may be further complicated by other infections [6]. Figure 1 summarizes the geographic distribution of the *hvKp* infection [65,66,67,68,69].

Current research is unsure of the exact mechanisms through which *hvKp* spreads through communities; however, based on data from the cKP bacteria, probable vectors may include contaminated water or food, direct transmission between close personal contacts, or animal-to-human transmission [65]. In support of these routes of acquisition, an investigation of ready-to-eat vegetables identified the *hvKp* strain, positive for the “string test”, isolated from cucumber [70]. Additionally, *hvKp* strains have been isolated from public water environments in Brazil, implicating contaminated water as another source of acquisition of these bacteria [71]. The gastrointestinal carriage is regarded as a major source of infection, especially in intensive care patients [72]. Investigation of fecal bacteria samples from healthy and *K. pneumoniae* liver abscess (KLA) patients showed similar bacterial serotypes and genotypes [73]. Interestingly, a case report from Japan showed that the stool cultures from two healthy relatives of KLA patients were *hvKp*-positive, rendering the relatives carriers [74]. These two reports suggest that the gut is also a reservoir for *hvKp,* and as proposed by Zhu J. et al. [10], this is plausible as this could be the passageway through which *hvKp* spreads from the intestinal barrier to the liver, causing KLA.

Several factors stand behind the higher incidence of infections caused by *K. pneumoniae* compared to other Gram-negative opportunistic pathogens, including bacteria’s ability to tolerate starvation [75], inherent antibiotic resistance [76], outcompete other bacteria [77], voluntarily exchange DNA with other human microbiome bacteria [78], and acquired antibiotic resistance and virulence-enhancing genes [79].

Antibiotics are diverse chemical substances that play a crucial role in treating and controlling the spread of infectious diseases. Two mechanisms exist through which antimicrobial drugs act on bacteria, either via a bacteriostatic or bactericidal mode of action, which hinders bacterial replication or kills the bacteria, respectively [29]. One of the recent and major challenges to public health is antimicrobial resistance (AMR). AMR is defined as the microorganisms’ ability to live and thrive in the presence of antimicrobial agents, a phenomenon associated with increased morbidity and mortality rates [80]. Bacterial resistance can be divided into three patterns: MDR (multidrug-resistant), where bacteria are resistant to more than one antimicrobial agent; XDR (extensively drug-resistant), refers to bacteria that remain susceptible to only one to two antimicrobial categories; PDR (pan-drug resistance) which includes bacteria that are resistant to all agents in all antimicrobial categories [81]. Classical *K. pneumonia* (cKP) has a natural resistance against certain antibiotics, such as ampicillin, carbenicillin, and ticarcillin, due to the production of an enzyme known as chromosomal penicillinase, sulfhydryl variable (SHV-1) [82]. However, third- and fourth-generation cephalosporins, quinolones, or carbapenems are effective antimicrobial drugs for treating *K. pneumonia* [83], making cKP a non-concerning pathogen. Two major types of antibiotic resistance were frequently found in *K. pneumoniae* infections. The first mechanism involves the production of the extended-spectrum beta-lactamase (ESBL) enzyme, which acts on beta-lactam antibiotics, including penicillin, cephalosporins, and monobactams. Thus, ESBL-producing *K. pneumoniae* is rendered resistant to such antibiotics [33]. Today, MDR ESBL-producing cKP are among the pathogens most associated with nosocomial infections [82]. Carbapenem drugs such as imipenem and meropenem are still the “gold standard” therapy for treating serious and invasive ESBL infections and are linked to better outcomes in patients with severe infections. In addition to carbapenems, β-lactam/β-lactamase inhibitor (BLBLI) combinations such as piperacillin–tazobactam (PTZ) are also found to be effective in treating ESBL-producing *K. pneumonia* [84]. The second mechanism is more concerning, through which *K. pneumonia* will develop resistance to almost all available beta-lactams, including carbapenems. This resistance is obtained by the help of carbapenemase enzymes that are capable of hydrolyzing carbapenems. These types of bacteria are referred to as CRKP, which is short for carbapenem-resistant *Klebsiella pneumonia* [33]. There are indications that, in certain conditions, combination therapy is advantageous. In accordance with some studies, colistin is best taken in combination with another antibiotic, although the effectiveness of this treatment is still debatable [85].

A Kp isolate was considered *hvKp* when at least three or more of the following virulence genes were detected with >99% full-length gene coverage: *iucA*, *iroB*, *peg-344*, *rmpA*, and *rmpA2* [19]. Moreover, both cKP and *hvKp* possess type 1 (mannose-sensitive) and type 3 (mannose-resistant) fimbriae. In cKP strains, these fimbriae have been shown to adhere to host epithelial cells from the respiratory and urinary tracts and add to infection. Although little work has been conducted on *hvKp*, a recent study examined the regulation of type 3 fimbriae in the *hvKp* strain CG43. This study confirmed observations that type 3 fimbriae contribute to biofilm formation and demonstrated that expression positively correlated with iron concentration [6]. 

## 5. Siderophores

Iron is an indispensable element for bacteria growth both in vitro and in vivo. Out of the 12 identified iron transport systems in *K. pneumoniae*, three siderophore-based systems (Yersiniabactin [86], Iuc [87], and IroA [88]) are required for complete *K. pneumoniae* virulence. In mammalian cells, free iron is very scarce due to nutritional immunity metal deprivation by the host, where it is stored bound to proteins and other molecules. Thus, during host infection, *K. pneumoniae* deals with this iron shortage by synthesizing small iron-scavenging molecules called siderophores.

### 5.1. Aerobactin

**Figure 2:**
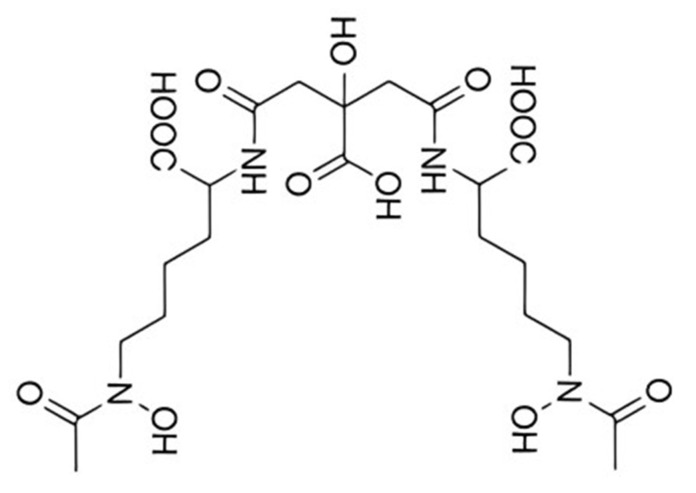
The structure of aerobactin.

Many pathogenic Gram-negative bacteria produce aerobactin, a citrate-hydroxamate siderophore (Figure 2) that was first identified about 50 years ago [88]. As mentioned earlier, it has recently been shown that aerobactin is essential for mediating the increased pathogenicity of a particularly invasive strain of *K. pneumoniae* (*hvKp*).

#### 5.1.1. Aerobactin Biosynthesis

Four proteins, namely IucA, IucB, IucC, and IucD, work together to biosynthesize the hydroxamate-based siderophore. These proteins are encoded by four genes, *iucA*, *iucB*, *iucC*, and *iucD*, which are part of the aerobactin biosynthetic operon. Firstly, hydroxylase IucD and acetyltransferase IucB work in conjunction to create N^6^-acetyl-N^6^-hydroxylysine (ahLys). The N^6^-hydroxylation of L-lysine to N^6^-hydroxy-L-lysine (hLys) is catalyzed by monooxygenase IucD. Then, N^6^-acetyl-N^6^-hydroxy-L-lysine (ahLys) is produced when acetyltransferase IucB transfers an acetyl from acetyl CoA to hLys. The main carboxylates of citrate are subsequently joined by two molecules of ahLys. Initially, one ahLys molecule is stereo-specifically added to a primary carboxylate of citrate by the aerobactin synthase IucA. A second aerobactin synthetase, IucC, then adds the second ahLys molecule to produce aerobactin [89,90]. IucA and IucC are members of an ATP-dependent ligase family that produces a variety of siderophores, including those linked to virulence [91]. Hydroxamate-based siderophores are referred to as non-ribosomal peptide synthetase-independent siderophores (NISs) [92].

**Figure 3:**
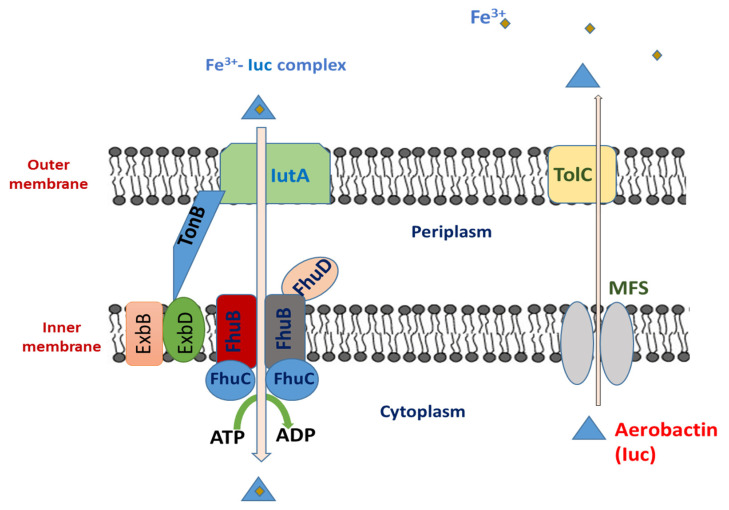
Aerobactin transport through the inner and outer membranes of *K. pneumoniae*.

After synthesis, aerobactin is released to the external environment by the MFS and TolC transporters, which transport aerobactin into the periplasm and to the external environment, respectively. Then, via TonB-dependent manner, the iron-loaded aerobactin is transported into the periplasm via the outer membrane receptor IutA [90]. The latter enhances the transport of iron-aerobactin complexes into the periplasmic region, where FhuB, FhuC, and FhuD mediate the internalization into the cytoplasm. Since these five proteins are involved in many transport systems, they are not regarded as components of the aerobactin system [93]. The export and import of aerobactin is illustrated in Figure 3.

#### 5.1.2. Genetic Regulation

The proteins IucD, IucB, IucA, and IucC, which are responsible for aerobactin production, are expressed by the operon (*iucABCD*). The concentration of intracellular iron Fe^2+^ controls the expression of these genes and, consequently, the production of this siderophore; low intracellular Fe^2+^ induces the production of aerobactin by the bacterium, while high or sufficient intracellular levels of Fe^2+^ suppress the production [94].

In fact, when the iron-loaded siderophore is transported into the cell, Fe^2+^, by acting as a corepressor, interacts with the DNA-binding protein Fur and blocks the expression of genes that promote iron acquisition [95]. Thus, Fur/Fe^2+^ acts as a negative regulator for *iucABCD* transcription [96].

### 5.2. Enterobactin

**Figure 4:**
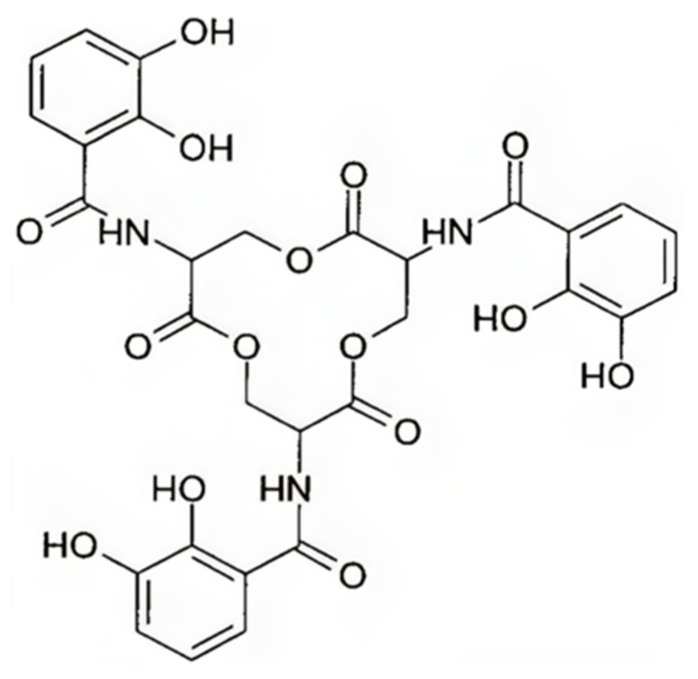
The structure of enterobactin.

Enterobactin (Ent), also known as enterochelin, is the primary siderophore of the form catechol produced by *K. pneumoniae* (Figure 4).

#### 5.2.1. Enterobactin Biosynthesis

The chorismic acid-dependent production of enterobactin involves the operon *entABCDEFH*. The two-module non-ribosomal peptide synthetase (NRPS), which consists of EntE, EntB, and EntF, assembles Ent. Ent is a 2,3-dihydroxybenzoylserine trilactone formed from 2,3-dihydroxybenzoic acid (DHB) and serine [97]. EntD catalyzes the post-translational 4′-phosphopantetheinylation of EntB and EntF, which is necessary for the covalent anchoring of assembly line intermediates. Meanwhile, EntC, EntB, and EntA sequentially redirect the major metabolite chorismate to DHB [98,99]. After being generated in the cytoplasm, Ent crosses the inner membrane by EntS, a prominent facilitator-subfamily exporter. It is then exported through the outer membrane by a TolC-dependent mechanism [100,101]. After chelating iron, the complex (Fe^3+^-Ent) is taken up by the TonB-dependent outer-membrane Ent-specific porin, FepA [102,103], assisted by FepB through the periplasm, and pumped into the cytoplasm by FepC-catalyzed ATP hydrolysis [104] through the two-protein inner-membrane channel FepDG. The esterase activity of the protein Fes releases Fe^3+^ from Ent before the tightly bound ferric iron can be transferred to intracellular iron transporters [105].

#### 5.2.2. Genetic Regulation

The transcription of the entire Ent system is controlled by the same iron-dependent DNA-binding transcription repressor Fur, which acts as a sensor for intracellular iron; it separates from DNA when iron levels are low [106]. Consequently, it stimulates the transcription of Ent synthesis, export, and import genes. Conversely, under high intracellular iron levels, Fur binds to its corresponding DNA sequence, blocking the transcription of Ent-related genes.

### 5.3. Salmochelin

**Figure 5:**
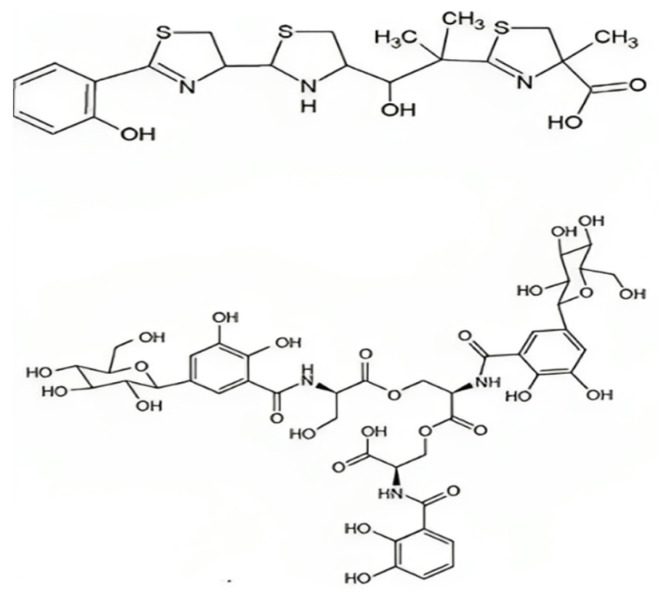
The structure of yersiniabactin and salmochelin.

Salmochelin (Figure 5) represents a glycosylated version of Ent [107]. The *iroA* gene and *iroBCDE* gene cluster are responsible for this alteration [87]. IroB is a glycosyltransferase, IroD is comparable to the enterobactin esterase Fes, and IroN resembles the enterobactin receptor FepA [108]. IroB is the glucosyl transferase that binds the glucose moieties to enterobactin, whereas IroC is thought to be an inner membrane transporter that facilitates the export of apo siderophores [109]. Meanwhile, IroN mediates the transport of the iron-loaded form of salmochelin into the bacteria [110]. The *ent* genes are organized into six clusters, each including three sets of divergent promoters. Numerous Fur binding sites are present in these promoter regions, and iron negatively controls the operons’ expression [111]. In fact, there are about 10-sequence binding sites for Fur overlap in the *iroBCDE* operon [112] and the *iroN* [113]. Through which Fur can bind and repress transcription.

### 5.4. Yersiniabactin

Non-ribosomal peptide synthetase (NRPS) and polyketide synthase (PKS) work together to carry out the synthesis of yersiniabactin (Ybt). Salicylate, three cysteines, a linker malonyl group, and three methyl groups from S-adenosylmethionine are the building blocks of Ybt [114], as shown in Figure 4. The synthesis of Ybt involves seven proteins: YbtD, YbtE, YbtS, YbtT, and YbtU, in addition to two high-molecular-weight proteins 1 and 2. Through the coenzyme, A moiety 4′-phosphopantetheine, which is supplied to the carrier protein domain sites of these enzymes by YbtD phosphopantetheinyl transferase, the active components of Ybt are attached to HMWP1 and HMWP2. YbtS synthesizes salicylate from chorismate; YbtE transfers it to HMWP2 and activates it by adenylation. The Ybt synthesis frame scaffold is provided by HMWP1 and HMWP2. Two cysteines are cyclized and condensed by the NRPS enzymatic domains found in HMWP2, resulting in the formation of two thiazoline rings that are attached to the salicylate component [115,116]. Two NRPS domains of HMWP1 cyclize and condense the third cysteine molecule that makes up the last thiazoline ring. YbtU converts the middle thiazoline ring to thiazolidine. The resulting siderophore is then released by the terminal HMWP1 thioesterase domain. It is believed that abnormal molecules are eliminated from the enzyme complex by the presumed type II thioesterase YbtT [86,117]. It is believed that the transporters required for the secretion of this siderophore are encoded by the *ybt* and *fyu* genes, while the uptake receptor is expressed from the *ybtQ* gene. The proteins necessary for yersiniabactin production are encoded by the *irp* genes [118]. The outer membrane receptor is FyuA, and YbtPQ constitutes the ABC transporter in the cytoplasmic membrane.

### 5.5. Siderophores and Virulence

*K. pneumoniae* produces several siderophores to maximize its colonization of various tissues and provide substitutes in case the host neutralizes one [119,120]. Aerobactin has the lowest affinity for iron among these siderophores, while enterobactin has the highest affinity [121,122]. Enterobactin expression is practically universal in both classical and *hvKp* strains, but the expression of the other siderophores is less conserved. As a result, enterobactin is thought to be the main siderophore for iron uptake used by *K. pneumoniae* [87,123]. Lipocalin-2, a substance released by the host, neutralizes enterobactin [86,108]. During infection, lipocalin-2, a multifunctional protein with several antimicrobial properties, is secreted from a variety of cell types, including neutrophils. Although it is produced at a basal level, the host increases the transcription of this factor in response to a respiratory tract infection caused by *K. pneumoniae* [124,125]. Instead of killing *K. pneumoniae*, lipocalin-2 stops it from growing by attaching to and neutralizing some of its produced siderophores, which prevents *K. pneumoniae* from scavenging iron from the host [126]. Additionally, lipocalin-2 has proinflammatory properties. When the host produces more of it, neutrophil recruitment to the bacterial infection site increases significantly, most likely because of IL-8 production [16,127]. In the absence of lipocalin-2, enterobactin aids in lung colonization and spread [128]. However, *K. pneumoniae* strains that solely generate this siderophore are eliminated in the presence of lipocalin-2 [129].

Remarkably, yersiniabactin was detected in 90% of *hvKp* clinical isolates but only in approximately 18% of the classical isolates [123,128]. Nonetheless, it is overrepresented in respiratory tract isolates of *K. pneumoniae*, along with enterobactin [129]. Significantly, yersiniabactin is expressed during lung infection, and lipocalin-2 does not suppress its action in vivo during the early stages of lung infection. This is probably because its structure differs greatly from enterobactin [108,129]. This facilitates the growth of *K. pneumoniae* during infection to large bacterial burdens in the lungs [129]. Although yersiniabactin does not appear to be affected by lipocalin-2, it cannot obtain the iron needed for *K. pneumoniae* to proliferate when transferrin, a host protein, is present [129]. The reason for this is that strains that solely express the yersiniabactin siderophore are unable to spread from the lungs. This is probably due to transferrin’s ability to inhibit *K. pneumoniae* development in blood as it is concentrated in blood plasma. Hence, infection by *K. pneumoniae* strains that solely generate yersiniabactin is probably not fatal for immunocompetent people [129]. Research also indicates that yersiniabactin might be involved in the pathophysiology of *hvKp* infections in mice, which are used as a model for liver abscesses but not in pneumonic models [123,130].

As an evolutionary escape mechanism, the C-glucosylation of enterobactin produces salmochelin. This structural alteration stops lipocalin-2 from binding salmochelin, thus, inhibiting its neutralization and the production of inflammation that is dependent on lipocalin-2 [14]. Therefore, salmochelin’s ability to promote *K. pneumoniae* colonization of the nasopharynx in a lipocalin-2-sufficient host is unsurprising [16]. One can assume that patients infected with salmochelin-positive strains may be less immunocompromised overall and that salmochelin-producing strains are more virulent because the expression of salmochelin permits nasopharyngeal colonization by *K. pneumoniae* in hosts capable of producing lipocalin-2. Salmochelin is only found in 2–4% of nosocomial *K. pneumoniae* strains, which is consistent with this assumption. However, it is significantly more common in *hvKp* strains, where more than 90% of the *hvKp* strains that are linked to pyogenic liver abscesses release salmochelin [122,123,129].

Concerning aerobactin, the citrate-hydroxamate siderophore is present in 93 to 100% of *hvKp* isolates; it is rarely expressed by classical nosocomial *K. pneumoniae* clinical isolates, as it is only identified in approximately 6% of classical strains [17,119,121]. Aerobactin is usually linked to a hyper-capsule, even though not all strains with hyper-capsules have this siderophore [17]. This correlation results from the co-expression of the aerobactin transporter, *iutA*, and the aerobactin gene cluster, *iucABCD*, on the same virulence plasmid as *rmpA*, an enhancer of capsule synthesis [17,131]. It is interesting to note that while aerobactin is uncommon in conventional strains that cause lung infections, it might be the main siderophore secreted in some *hvKp* strains that cause lung infections [128]. Aerobactin makes up the great majority of the siderophores produced in at least one *hvKp* strain, which possess an enhanced capacity to generate iron through amplified siderophore production [132]. Additionally, in pneumonic and subcutaneous mice infection models, aerobactin is required for effective infection by *hvKp*, but not enterobactin, yersiniabactin, or salmochelin [133]. Aerobactin functions in a redundant manner alongside yersiniabactin and salmochelin in a mouse model of intraperitoneal *hvKp* infection; the strain is only inhibited in vivo by simultaneous deletion of all three siderophores [33].

### 5.6. Heme Uptake System hmuRSTUV

Erythrocyte hemoglobin, which is made up of four subunits with an iron atom attached to each heme group, contains the majority of the host iron. This is an excellent source of iron that siderophores are unable to access. The adult erythrocyte circulates for around 120 days until phagocytes, primarily found in the liver, bone marrow, and spleen, absorb and disintegrate it. Iron is then liberated when heme is liberated from the phagolysosomes and broken down by heme oxygenase-1. By creating high-molecular-weight haptoglobin–hemoglobin complexes, haptoglobin eliminates cell-free hemoglobin (CFH) from the plasma, preventing the toxicity that CFH causes. After haptoglobin’s ability to bind hemoglobin is exceeded, unbound hemoglobin can release heme directly into the circulation [134,135]. The oxidized form of heme is called hemin. By using the HmuRSTUV hemin absorption mechanism, *K. pneumoniae* can scavenge heme/hemin as an iron source. HmuR is an outer membrane receptor that requires TonB, just like siderophore receptors, in order to utilize heme. Hemin is transported into the cytoplasm by HmuTUV, an inner membrane ABC transporter, whereas HmuS is necessary for hemin breakdown and iron release [136].

## 6. Mortality and Morbidity of *K. pneumonia*

A systematic review and meta-analysis performed by Li et al. concerning *Klebsiella pneumoniae* bacteremia mortality showed 17% of deaths at 7 days, 24% at 14 days, 29% at 30 days, 34% at 90 days, and 29% in hospitals. Significantly higher 30-day mortality rates were linked to more than 50% of *K. pneumoniae* cases that were hospital-acquired (HA), extended-spectrum beta-lactamase (ESBL), carbapenem-resistant (CR), and intensive care unit (ICU) [137].

Numerous studies have shown that a number of comorbidities, such as diabetes, cancer, chronic liver disease, and biliary tract disease, are risk factors for KP-bacteremia. More attention should be paid to KP-BSI (secondary KP bloodstream infection), as it deteriorates the prognosis of patients with KP pneumonia [138].

A conservative estimate of the direct medication cost per patient infected with *K. pneumoniae* carbepemase is nearly USD 4100, with approximately 60% of expenses incurred during the illness. KPC presents an economic risk to the healthcare industry as a whole. The prevalence of these germs is rising globally, and most health systems are finding it increasingly difficult to handle this load. Developing new antimicrobials and regimens is essential for treating KPC, with the goal of reallocating resources to improve the efficacy of medical care [139,140].

## 7. Conclusions

*Klebsiella pneumoniae* is a Gram-negative bacterial pathogen that is becoming more significant and can lead to serious organ damage and even death. Currently, there are two pathotypes of *K. pneumoniae* that are known as classical *K. pneumoniae* (cKp) and hypervirulent *K. pneumoniae* (*hvKp*), each of which poses different difficulties for clinicians. Both pathotypes are common worldwide, but during the past three decades, the nations that make up the Asian Pacific Rim have seen a steady rise in the incidence of infections caused by *hvKp*. In contrast, infections caused by *hvKp* are becoming more widely known outside Asia, whereas cKp has historically been the most common offending agent in Western nations. Virulence factors that are necessary for survival and pathogenicity include capsules, siderophores, lipopolysaccharides, fimbriae, outer membrane proteins, and type 6 secretion system, of which the first two are dominant. Siderophores are high-affinity, low-molecular-weight iron chelators that are essential for bacterial pathogenicity and proliferation. The particular mixture of siderophores that *K. pneumoniae* secretes during infection can affect host survival, systemic dispersion, and tissue localization. Specifically, gene clusters that encode the production of the siderophores salmochelin (*iro*) and aerobactin (*iuc*) are linked to invasive illness and are frequently seen in hypervirulent *K. pneumoniae* clones that cause serious community-associated diseases, such as pneumonia and liver abscess. However, reports of *iuc* in MDR strains in a hospital context are equally concerning.

## Data Availability

No data was reported in this study.
